# Conserved CD4 T-cell responses correlate with antibody neutralization in solid organ transplant recipients after bivalent SARS-CoV-2 vaccination

**DOI:** 10.3389/fimmu.2026.1856861

**Published:** 2026-07-24

**Authors:** Georgia Stavrakis, Katerina Roznik, Laila Stoddart, T. Scott Johnston, Snigdha Panda, Camille Hage, Aura T. Teles, Yolanda Eby, William A. Werbel, Aaron A. R. Tobian, Aaron M. Milstone, Alessandro Sette, Alba Grifoni, Andrew H. Karaba, Elizabeth A. Thompson, Andrea L. Cox

**Affiliations:** 1 Department of Molecular Microbiology and Immunology, Johns Hopkins Bloomberg School of Public Health, Baltimore, MD, United States; 2 Department of Medicine, Johns Hopkins University School of Medicine, Baltimore, MD, United States; 3 Department of Surgery, Johns Hopkins University School of Medicine, Baltimore, MD, United States; 4 Department of Pathology, Johns Hopkins University School of Medicine, Baltimore, MD, United States; 5 Department of Pediatrics, Johns Hopkins University School of Medicine, Baltimore, MD, United States; 6 Center for Vaccine Innovation, La Jolla Institute for Immunology (LJI), La Jolla, CA, United States; 7 Department of Pathology, University of California, San Diego, San Diego, La Jolla, CA, United States

**Keywords:** COVID-19, immunocompromised, memory, T cells, vaccines

## Abstract

**Introduction:**

Vaccines against SARS-CoV-2 and influenza require reformulation due to viral evolution. It remains unclear how vaccination against evolving antigens influences T-cell responses and the impact of reformulation on T-cell responses to new variants in immunocompromised hosts. Solid organ transplant recipients (SOTRs) had significantly lower antibody levels following vaccination and required repeated boosting during the COVID-19 pandemic.

**Methods:**

With the introduction of the Omicron spike (S) to the bivalent mRNA vaccines in 2022, the relative contribution of T-cell responses cross-reactive for Omicron mutated S sequences was assessed via intracellular cytokine staining using peptide pools containing conserved or mutated S sequences.

**Results:**

S-specific CD4 T cells recognize both conserved and Omicron mutated S sequences pre- and post-bivalent vaccination, even in the absence of evidence of prior infection as detected by T-cell responses to a novel peptide pool. CD4 T-cell responses to both conserved and Omicron mutated sequences correlated with antibody pseudo-neutralization of BA.5 S. Importantly, pre-bivalent conserved S CD4 T-cell responses significantly correlated with antibody pseudo-neutralization of BA.5 S post-bivalent.

**Discussion:**

These results emphasize the importance of conserved and cross-reactive responses in vaccine immunogenicity, providing a mechanism through which repeated boosting enhances vaccine immunogenicity in SOTRs and other immunocompromised populations.

## 1 Introduction

Viral variation is a barrier to effective vaccine development against multiple pathogens and viral evolution necessitates reformulation of vaccines against viruses like influenza and SARS-CoV-2, the virus that causes COVID-19. Antibody pressure drives global evolution of SARS-CoV-2, prompting reformulations of vaccines designed to improve immune responses against emergent variants (1). However, there is evidence that in addition to supporting antibody responses, T-cell responses play an important role in vaccine protection against severe COVID-19, particularly against variants that partially escape antibodies (2). The role of vaccine-primed T cells cross-reactive between variants is particularly unclear for immunocompromised populations (3–8). Generally, immunocompromised individuals develop weaker immune responses to vaccination and maintain an increased risk of severe infection (7–13). Solid organ transplant recipients (SOTRs) are particularly at-risk for infections, with reduced immune responses to most vaccines, and vaccine-preventable diseases remain a significant cause of morbidity and mortality in this population (14–18). Ongoing risks relate in part to immunosuppressive medications used to prevent organ rejection post-transplant; hence, the primary approach for immunizing SOTRs has been to immunize prior to organ transplantation (7, 19). While it has been shown that various immunosuppressive medications significantly impair T-cell effector function *in vitro* (19–22), memory immune responses generated prior to organ transplantation can be boosted post-transplantation upon re-immunization (12).

Because of low seroconversion rates, SOTRs were recommended to receive a third dose of monovalent mRNA vaccines encoding ancestral Spike (S) before additional booster doses were recommended for the general population (23–25). SOTRs were also recommended to receive a dose of bivalent mRNA vaccines after Omicron became the dominant circulating variant (25, 26), though benefits of the updated formulations compared to ancestral vaccines and the contribution of conserved versus novel T-cell responses upon boosting remain unclear (27, 28).

Omicron sublineages have multiple changes in the S receptor binding domain (RBD), the critical binding site of neutralizing antibodies (1, 29–31). Despite SARS-CoV-2 evasion of neutralization, vaccines continued to protect people from severe disease, suggesting a role for cellular immunity in protection (32, 33). This could be due to the recognition of T-cell epitopes in regions of S other than RBD, where there are fewer sequence differences between ancestral and Omicron sublineages, providing a neutralizing antibody-independent means through which T cells could contribute to broadly protective immunity to SARS-CoV-2 variants (2, 34, 35). Whether immunocompromised individuals vaccinated post-immunosuppression generate *de novo* T cells to novel viral variants or simply expand pre-existing cross-reactive responses has not been fully explored (8, 36, 37), particularly in SOTRs, a high-priority population at high risk of severe infections. Additionally, what T-cell help is required to make variant neutralizing antibody responses is unclear. The COVID-19 pandemic offers a unique opportunity to understand how novel T-cell responses are generated in individuals on immunosuppressive medications (37–39). Understanding whether memory T-cell responses are expanded along with the production of *de novo* T cells will be relevant for the design of future vaccines for individuals on immunosuppressive drugs. Thus, we aimed to understand how bivalent mRNA immunization impacted S-specific T-cell responses in SOTRs who had previously received monovalent mRNA vaccines.

We found that S-specific CD4 T-cell responses are composed of responses to both conserved and Omicron mutated S sequences post-bivalent vaccination. Furthermore, we show that S-specific CD4 T cells from prior repeated boosting with monovalent vaccines respond to Omicron mutated S even prior to receiving the bivalent vaccine in SOTRs. Additionally, the presence of polyfunctional CD4 responses to conserved S epitopes pre-bivalent vaccination correlated with antibody responses to Omicron variant S in SOTRs post-bivalent vaccination. Consistent with the need to vaccinate SOTRs repeatedly to generate neutralizing responses to the ancestral SARS-CoV-2 viruses, these data suggest that T-cell responses to conserved or cross-reactive antigen sequences are important in SOTRs for generating responses to novel variants and should be considered in the design of vaccines against rapidly evolving pathogens.

## 2 Results

### 2.1 Bivalent mRNA immunization in SOTRs leads to increased S-specific antibody and polyfunctional CD4 T cells recognizing ancestral and Omicron S

The effects of bivalent boosting were assessed by comparing immune responses before and 2 weeks after bivalent immunization within a cohort of SOTRs described in **Table 1**. Humoral S-specific immunity was assessed using percent ACE2 inhibition by SOTR plasma, also referred to as pseudo-neutralization, which has previously shown to correlate with live virus neutralization (40). Bivalent mRNA immunization in SOTRs boosted humoral immunity in most participants against both ancestral and SARS-CoV-2 variants (**Supplementary Figure 1A**).

**Table 1:** Characteristics of SOTRs receiving bivalent SARS-CoV-2 vaccine.

*N* = 24	
**Age, median [IQR]**	62 [47–68]
**Female**	14 (58%)
**White race**	23 (96%)
Organ transplanted	
Kidney	14 (58%)
Liver	5 (21%)
Lung	3 (12%)
Multi-organ	2 (8%)
**Years since transplant, median [IQR]**	4.5 [2.4–10.7]
Immunosuppressants	
Anti-metabolite^	18 (75%)
Calcineurin inhibitor	20 (83%)
mTOR inhibitor	5 (21%)
Steroids	15 (63%)
Triple immunosuppression*	10 (42%)
**Prior COVID-19 vaccines, median [IQR]**	4 [4–5]
**Prior COVID-19 infection reported**	8 (33%)
Omicron era	5 (21%)

^MMF, Mycophenolate mofetil; MPA, mycophenolic acid, or azathioprine.

*Triple immunosuppression defined as steroid, calcineurin inhibitor, and anti-metabolite.

**Figure 1:**
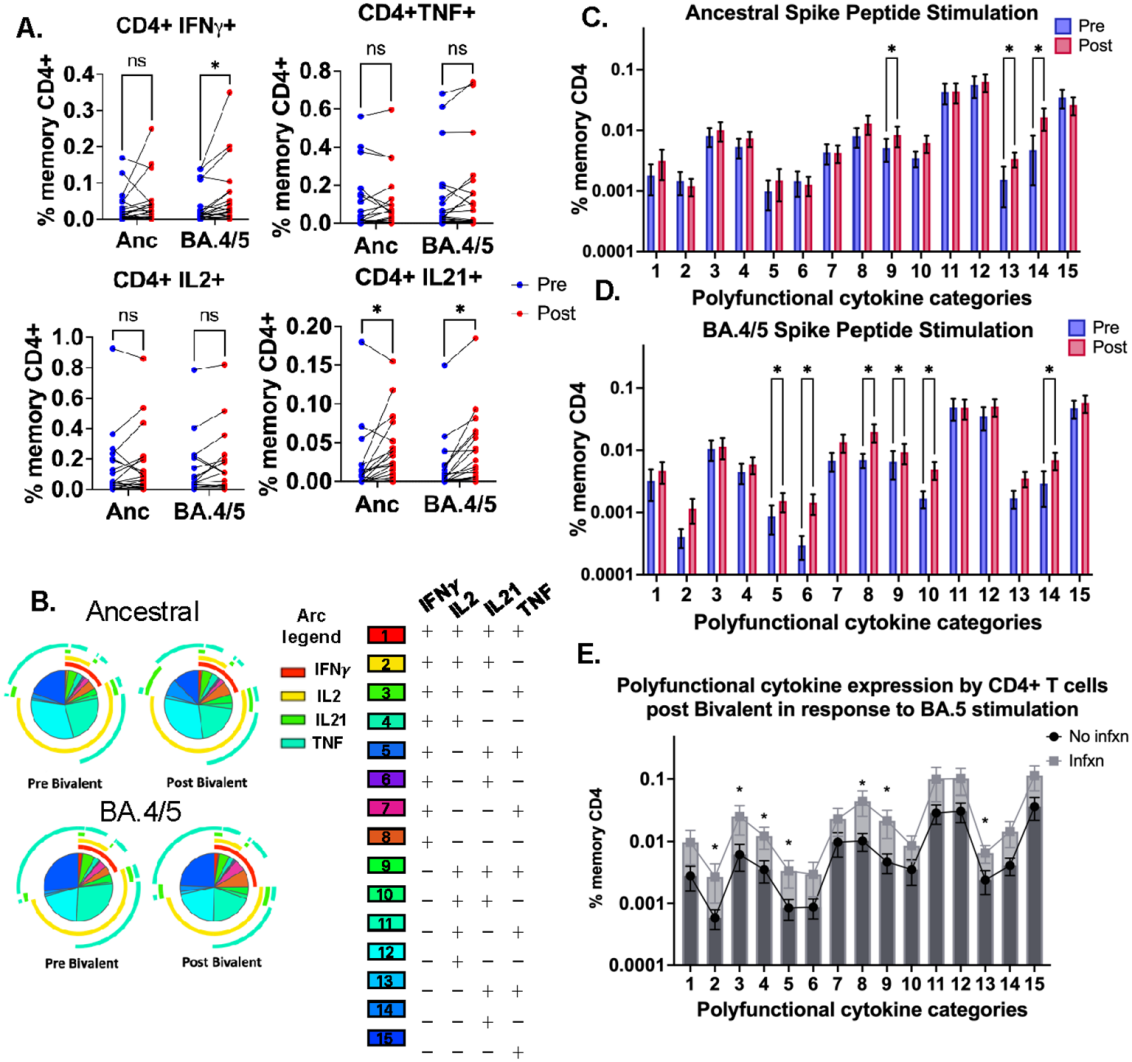
Bivalent immunization significantly increases the frequency of polyfunctional and effector-like CD4 T cells in response to Ancestral and Omicron spike. **(A)** Frequency of memory CD4^+^ T cells that produce cytokine (TNF, IFN-γ, IL-2, or IL-21) in response to ancestral (Anc) or BA.4/5 Spike (S) peptides. Paired comparison of S-specific CD4^+^ T-cell responses by pre- and post-bivalent vaccine stratified by stimulation with ancestral or BA.4/5 S peptides. **(B)** Pie charts show the fraction of total cytokine response comprising any combination of IFNγ, IL-2, TNF, or IL-21. Pie arcs show the proportion of CD4^+^ T cells producing each cytokine. **(C)** Frequency of polyfunctional cytokine categories in memory CD4^+^ T cells in response to overlapping Anc peptide pools. **(D)** Frequency of polyfunctional cytokine categories in memory CD4^+^ T cells in response to BA.4/5 S peptide pools. Significance tested using Wilcoxon matched-pairs signed rank test. **(E)** Frequency of polyfunctional cytokine categories in memory CD4^+^ T cells in response to BA.4/5 S peptide pools stratified by evidence of previous infection represented in a superimposed bar graph. Significance tested using multiple Mann–Whitney tests. *p < 0.05. Graphed frequencies are background subtracted from unstimulated conditions. Data shown as mean with SEM.

CD4 T cells and their T-cell helper subsets are instrumental for mediating downstream humoral responses (41–43). Given the necessity of CD4 T cells in generating robust antibody responses, S-specific CD4 T cells were assessed for quantitative cytokine expression in response to overlapping S peptide pools from ancestral (Anc) and Omicron (BA.4/5) SARS-CoV-2 vaccine antigens. Frequencies of cytokine-producing cells were determined from memory CD4 T cells after stimulation with Anc or BA.4/5 (**Supplementary Figure 1B**). We observed a significant increase in the frequency of interferon-gamma (IFNγ)-producing S-specific CD4 T cells following bivalent immunization in response to BA.4/5, but not to Anc stimulation (**Figure 1A**). In contrast, the frequencies of tumor necrosis factor (TNF)- and interleukin (IL)-2-expressing CD4 T cells were not significantly different post-bivalent immunization in response to either Anc or BA.4/5 stimulation (**Figure 1A**). Both Anc- and BA.4/5-stimulated CD4 T cells showed statistically increased frequencies in IL-21 expression (**Figure 1A**).

To further investigate the effects of bivalent boosting on the quality of the S-specific CD4 T-cell responses in SOTRs, we assessed polyfunctionality pre- and post-immunization after Anc or BA.4/5 stimulation (**Figure 1B**). Polyfunctionality refers to the ability of T cells to express multiple cytokines in response to a specific antigen and is a hallmark of protective immunity against pathogens (44–49). Ancestral S-specific CD4 T cells showed a significant increase in the frequency of CD4 T cells expressing IL-2^+^IL-21^+^TNF^+^ (category 9) and IL-21^+^TNF^+^ (category 13), and in monofunctional category IL-21^+^ (category 14) (**Figure 1C**; **Supplementary Figure 2A**). There was a significant increase in many more polyfunctional cytokine categories for BA.4/5 S-specific CD4 T cells comparing pre- to post-bivalent time points (**Figure 1D**; **Supplementary Figure 2B**). In addition to increased frequencies of categories 9 and 14 observed with Anc stimulation post-vaccination, there were statistically significant increases in frequencies of BA.4/5-stimulated S-specific CD4 T cells for polyfunctional cytokine categories 5, 6, 8, and 10 (**Figure 1D**; **Supplementary Figure 2B**). Generally, the Omicron-stimulated response after bivalent immunization was characterized by IFNγ and TNF polyfunctionality (**Figures 1B, D**).

In summary, bivalent immunization induced S-specific CD4 T cells with broadly polyfunctional and activated profiles in response to BA/4.5 stimulation compared to pre-vaccine time points. In contrast, S-specific CD4 T cells stimulated with Anc demonstrated a more cytokine-restricted profile and only had a significant increase in IL2^+^IL21^+^TNF^+^ and IL21^+^TNF^+^ polyfunctional T cells when comparing pre- and post-bivalent immunization.

### 2.2 Evidence of previous infection influences the magnitude, but not the quality, of the S-specific T-cell response

We observed high frequencies of cytokine expression in S-specific CD4 T in a subset of SOTR before bivalent boosting. We hypothesized that this might be due to previous SARS-CoV-2 infection, given previously published data from healthy individuals (50), and aimed to further assess the impact of previous infection on the observed differences between ancestral and Omicron S-specific T-cell responses. There were five reported Omicron-era infections in this cohort that occurred prior to receiving the bivalent vaccine; however, only four had sufficient PMBCs for this follow-up study. Interestingly, the highest frequencies of cytokine-expressing S-specific CD4 T cells were not found in those with previously reported infections. To detect previous unreported infections, we assessed T-cell responses to a non-S (not contained in the vaccine) peptide pool (NS), an assay previously shown to be more sensitive than anti-nucleocapsid ELISAs (51).

Cytokine expression from T cells stimulated with NS demonstrated evidence of six additional unreported previous infections within this SOTR cohort (**Supplementary Figure 3A**). Of the four reported infections within the cohort, T cells from only one SOTR had detectable cytokine responses to the NS peptide pool (**Supplementary Figure 3B**). However, T cells from the three individuals reporting infections without NS responses also failed to produce cytokines in response to S peptide pools despite previous immunization, indicating profound immunosuppression. Generally, SOTRs with T-cell evidence of infection but no clinical report of infection had higher frequencies of S-specific CD4 T cells (**Supplementary Figure 3B**), potentially indicating a correlation between T-cell responses and protection from symptomatic infection. Interestingly, SOTRs who did not report infection but had been infected based on the NS T-cell assay had relatively high frequencies of S-specific CD4 T cells, high pseudo-neutralization (PsNeut) of S, or a combination of both (**Supplementary Figure 3C**).

Interestingly, the SOTRs with the highest cytokine expression from S-specific CD4 T cells pre-bivalent boosting were those with evidence of previous infection based on responses to NS (**Supplementary Figure 3D**).

The impact of immunological evidence of previous infection on polyfunctionality pre- and post-bivalent vaccination was also assessed (**Supplementary Figures 4A–D**). For Anc- and BA.4/5-stimulated CD4 T cells, polyfunctional cytokine categories that significantly increased post-bivalent vaccination in the overall cohort remained trending or significantly increased in SOTRs without prior infection (**Supplementary Figures 4A,C**). In contrast, these differences were lost in SOTRs with evidence of previous infection (**Supplementary Figures 4B, D**), suggesting that infection had already augmented S-specific polyfunctional responses prior to bivalent immunization. We then further compared polyfunctionality post-bivalent vaccination between SOTRs without and with previous infection at the post-bivalent time point (**Figure 1E**). Although the magnitude of BA.4/5-specific polyfunctional responses differed by infection status, the relative composition of the polyfunctional response post-vaccination was similar between the groups (**Figure 1E**). In summary, polyfunctional cytokine responses to S were not further boosted by bivalent immunization if T-cell responses indicated prior infection. Additionally, evidence of previous infection generally influenced the magnitude rather than the qualitative polyfunctional profile measured post-bivalent immunization.

### 2.3 The S-specific CD4 T-cell polyfunctional cytokine response differs following Omicron and ancestral S peptide pools post-bivalent vaccination

**Figure 2:**
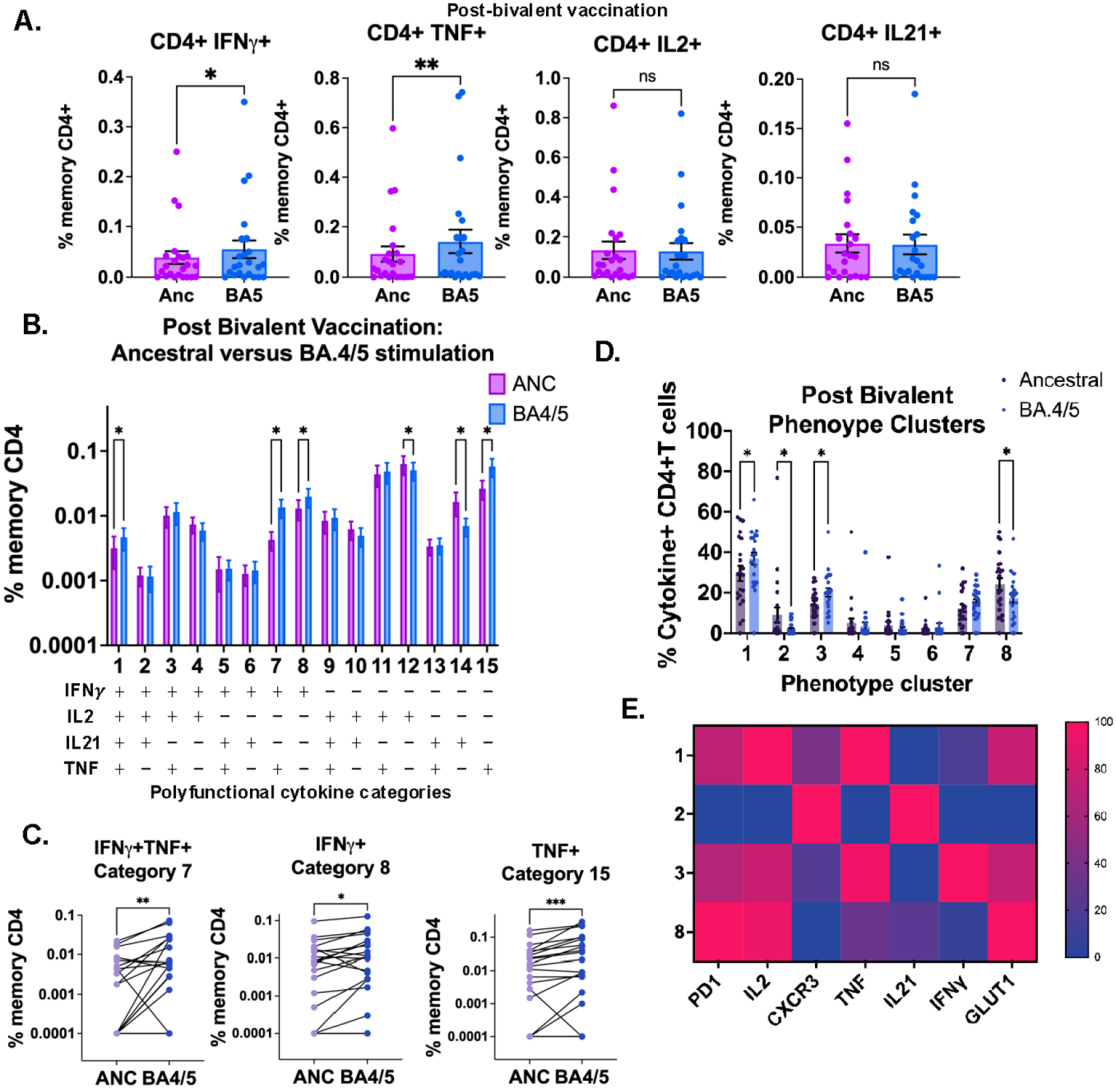
Bivalent vaccine boosts different S-specific CD4 T-cell phenotypes and polyfunctionality in response to ancestral and Omicron S peptide stimulation. **(A)** Frequency of memory CD4^+^ T cells producing cytokines (TNF, IFN-γ, IL-2, or IL-21) in response to ancestral (Anc) or BA.4/5 peptides at the post-bivalent time point. Sample paired comparison of CD4 responses recalled by Anc or BA.4/5 S peptides. All data shown as mean and SEM. **(B)** Frequency of polyfunctional cytokine categories in memory CD4^+^ T cells post-bivalent vaccination stratified by peptide stimulation. Data shown as mean with SEM. **(C)** Polyfunctional cytokine categories that are statistically significantly different post-bivalent vaccine in response to ancestral and BA.4/5 stimulation. Significance tested with Wilcoxon matched-pairs signed rank test. **(D)** Frequencies of clusters in ancestral and BA.4/5 S-specific CD4^+^ T cells post-bivalent vaccination. Significance tested using Wilcoxon matched-pairs signed rank test. *p < 0.05, **p < 0.01, ***p < 0.001. **(E)** Heatmap of MFIs for marker expression within unique phenotype clusters identified from S-specific CD4^+^ T cells.

We further investigated the boosting of the BA.4/5 S-specific CD4 T-cell responses by comparing Anc-stimulated responses to BA.4/5-stimulated responses at pre- and post-bivalent time points. When comparing Anc- to BA.4/5-stimulated CD4 T cells pre-bivalent immunization, only TNF-producing cells were significantly higher in BA.4/5 stimulation (**Supplementary Figure 5**). In contrast, in post-bivalent vaccination, there were significantly higher frequencies of IFNγ^+^ and TNF^+^ BA.4/5-stimulated S-specific CD4 T cells compared to Anc S-specific CD4 T cells (**Figure 2A**).

Prior to bivalent vaccination, categories 2 (IFNγ^+^IL-2^+^IL-21^+^) and 12 (IL-2^+^) were more abundant and category 15 (TNF^+^) was less abundant in Anc than in BA.4/5-specific CD4 T cells (**Supplementary Figure 6A**). Post-bivalent immunization, category 12 (IL2^+^) and category 14 (IL21^+^) remained more abundant in Anc-specific than BA.4/5-specific CD4 T cells. Interestingly, the polyfunctional response measured after bivalent vaccination in BA.4/5-specific CD4 T cells compared to Anc-specific responses was predominantly characterized by IFNγ and TNF, as evidenced by greater frequencies of combined production in category 7 (IFNγ^+^TNF^+^) and of monofunctional categories 8 (IFNγ^+^) and 15 (TNF^+^) compared to ancestral S responses (**Figures 2B, C**; **Supplementary Figure 6B**).

An additional 21 markers in our high-dimensional flow cytometry panel were used to further phenotype S-specific CD4 T cells identified by cytokine expression in response to S peptide stimulation (Anc or BA.4/5), through which we identified eight unique phenotypic clusters (**Supplementary Figures 6C, D**). Anc-stimulated CD4 T cells post-bivalent immunization had a greater frequency of phenotype clusters 2 and 8 compared to BA.4/5-stimulated responses (**Figure 2D**). Phenotype cluster 2 was characterized by high IL-21 and CXCR3 expression with lower expression of IFNγ, TNF, IL-2, PD-1, and Glut-1 (**Figure 2E**). Phenotype cluster 8 was characterized by high Glut-1, IL-2, and PD-1 expression in addition to low CXCR3 and IFNγ (**Figure 2E**). BA.4/5-stimulated CD4 T cells had higher frequencies of phenotype clusters 1 and 3 compared to Anc-stimulated responses (**Figure 2D**). Phenotype cluster 1 was characterized by low IL-21 and IFNγ and high IL-2 and TNF and cluster 3 by high TNF and IFNγ, and low IL-21 and CXCR3 expression (**Figure 2E**). PD-1 expression indicates T-cell activation (52, 53), and we have previously shown that Glut-1 expression increased upon activation in T cells from SOTRs (54), consistent with studies demonstrating that is it upregulated following TCR signaling (55). Therefore, these phenotypes complement the polyfunctional data and suggest that the TNF^+^ and IFNγ^+^ responses to BA.4/5 S are also associated with an effector, activated T-cell phenotype post-bivalent vaccination.

In summary, post-bivalent immunization, there were qualitative differences in the S-specific CD4 T-cell responses to Anc versus BA.4/5 stimulation. BA.4/5-specific CD4 T cells have increased IFNγ-expressing polyfunctionality and effector T-cell phenotypes post-bivalent immunization compared to Anc responses.

### 2.4 CD4 T-cell polyfunctionality in response to S can be attributed to both Omicron mutated and conserved S responses

**Figure 3:**
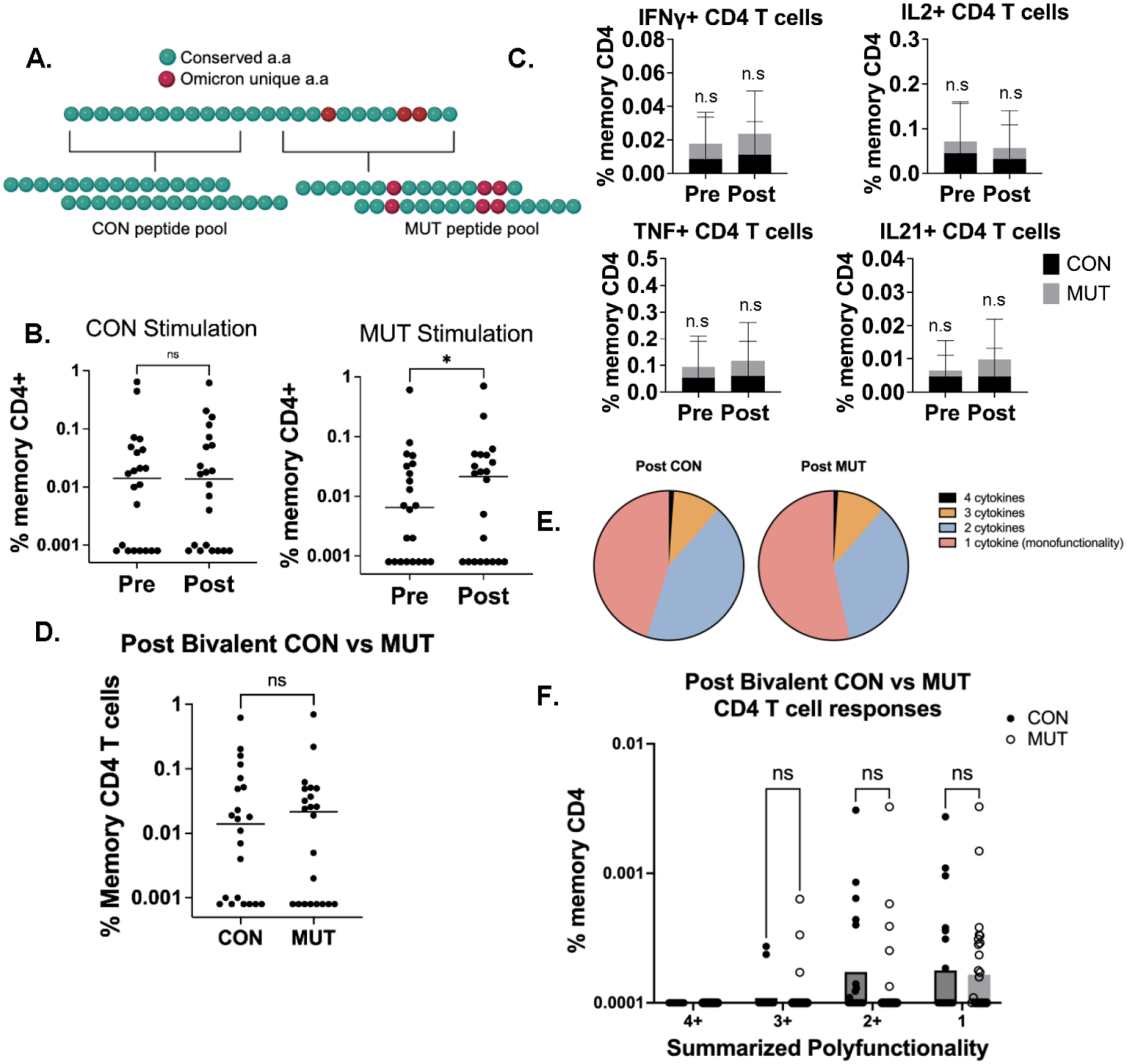
Post-bivalent vaccination, Conserved and Omicron mutated S-specific CD4 T-cell responses are similar. **(A)** Representation of Conserved (CON) and Omicron mutated (MUT) S peptide pools. **(B)** Frequency of bulk cytokine-producing memory CD4 T cells in response to CON and MUT peptide stimulation pre- and post-bivalent. *p < 0.05. **(C)** Frequency of memory CD4^+^ T cells producing cytokines (TNF, IFN-γ, IL-2, or IL-21) stratified by peptide pool stimulation pre- and post-bivalent. **(D)** Frequency of S-specific CD4 T cells post-bivalent vaccination in response to CON or MUT peptide stimulation with medians shown. Significance tested with Wilcoxon matched-pairs signed rank test. **(E)** Pie charts showing the summarized fraction of polyfunctional cytokine response comprising any four, three, two, or one cytokine from CD4 T cells post-bivalent. **(F)** Frequency of S-stimulated CD4 T-cell expression of four, three, two, or one cytokine in any combination post-bivalent. Significance tested with Wilcoxon matched-pairs signed rank test.

Given that most of the amino acid (a.a.) sequences are conserved between the Anc and Omicron S proteins, we determined if *de novo* priming occurs for CD4 T cells that recognize Omicron mutated S sequences or whether existing CD4^+^ T cells simply expand in response to conserved sequences. To address this question, we acquired peptide pools containing sequences conserved between Ancestral and BA.5 SARS-CoV-2 S (CON), or sequences containing mutations found in BA.5 (Omicron mutated/MUT), as previously described by Tarke et al. (2022) (56). Peptide pools for both conserved and Omicron mutated sequences consist of 15 a.a. peptides overlapping by 11 a.a. (**Figure 3A**). The Omicron mutated overlapping peptides pools encompass a.a. mutations with conserved flanking sequences. The conserved overlapping peptide pools only contain sequences with no Omicron a.a. mutations. Given the minimum length of CD4 T-cell epitopes, the recognized peptides in the MUT pool likely contain at least one mutation compared to ancestral sequence. Compared to the cytokine-positive T-cell frequencies observed in response to Ancestral and BA.4/5 overlapping S peptide pools, there were generally lower cytokine-positive T-cell frequencies observed in response to CON or MUT stimulation (**Supplementary Figure 7A**), consistent with a smaller pool size and fewer possible recognition sites within each pool. Frequencies of S-specific CD4 T cells in response to either CON or MUT stimulation were similar pre- and post-bivalent when looking at the expression of individual cytokines (**Figure 3B**). However, when comparing the total frequencies of CON- or MUT-specific T cells (i.e., the ability to make any cytokine in response to stimulation), there was a significant boost in the frequency of MUT-specific CD4 T cells post-bivalent compared to pre-bivalent (**Figure 3C**). Of note, MUT-specific CD4 T-cell responses were detected pre-bivalent vaccination, including in cases without evidence of previous infection (**Supplementary Figure 7B**), suggesting cross-reactivity with prior variants despite a lack of complete sequence identity. This is consistent with prior studies demonstrating that Omicron mutations, which are selected primarily to evade antibody responses, do not impair recognition of most commonly recognized ancestral S T-cell epitopes (56–61).

To assess differences in polyfunctionality with lower frequencies of cytokine-producing CD4 T cells, frequencies of CD4 T cells expressing any four, three, two, or one cytokine were compared rather than for individual polyfunctional categories (**Supplementary Figures 7C, D**). This grouped polyfunctionality was compared pre- to post-bivalent immunization for CON and MUT S peptide stimulation, with no significant differences measured (**Supplementary Figures 7C, D**).

We assessed if bivalent immunization resulted in quantitative differences between CON- and MUT-specific cytokine expression by CD4 T cells at the post-bivalent immunization time point. After bivalent immunization, there were no significant differences between the frequencies of cytokine-expressing CD4 T cells after CON S versus MUT stimulation, and this is consistent when comparing only cases with no evidence of previous infection (**Figure 3D**; **Supplementary Figure 8A**). Additionally, there are no significant differences in the frequencies of grouped polyfunctionality, indicating that CON- and MUT-specific CD4 T cells are similarly capable of polyfunctionality post-bivalent immunization (**Figures 3E, F**).

There is a quantitative boost in MUT-specific CD4 T cells post-bivalent vaccination compared to the frequency of MUT-specific CD4 T cells pre-bivalent. This boost in the MUT-specific response and the lack of differences between CON- and MUT-specific CD4 T cells post-bivalent suggest that qualitative differences observed in the polyfunctionality of the BA4.5 response in **Figure 2** can be attributed to responses to both cross-reactive, Omicron mutated and conserved spike sequences.

### 2.5 Conserved and Omicron mutated S-specific CD4 T cells post-bivalent immunization correlate with the ability of antibodies to neutralize ACE2 binding of BA.5 S

**Figure 4:**
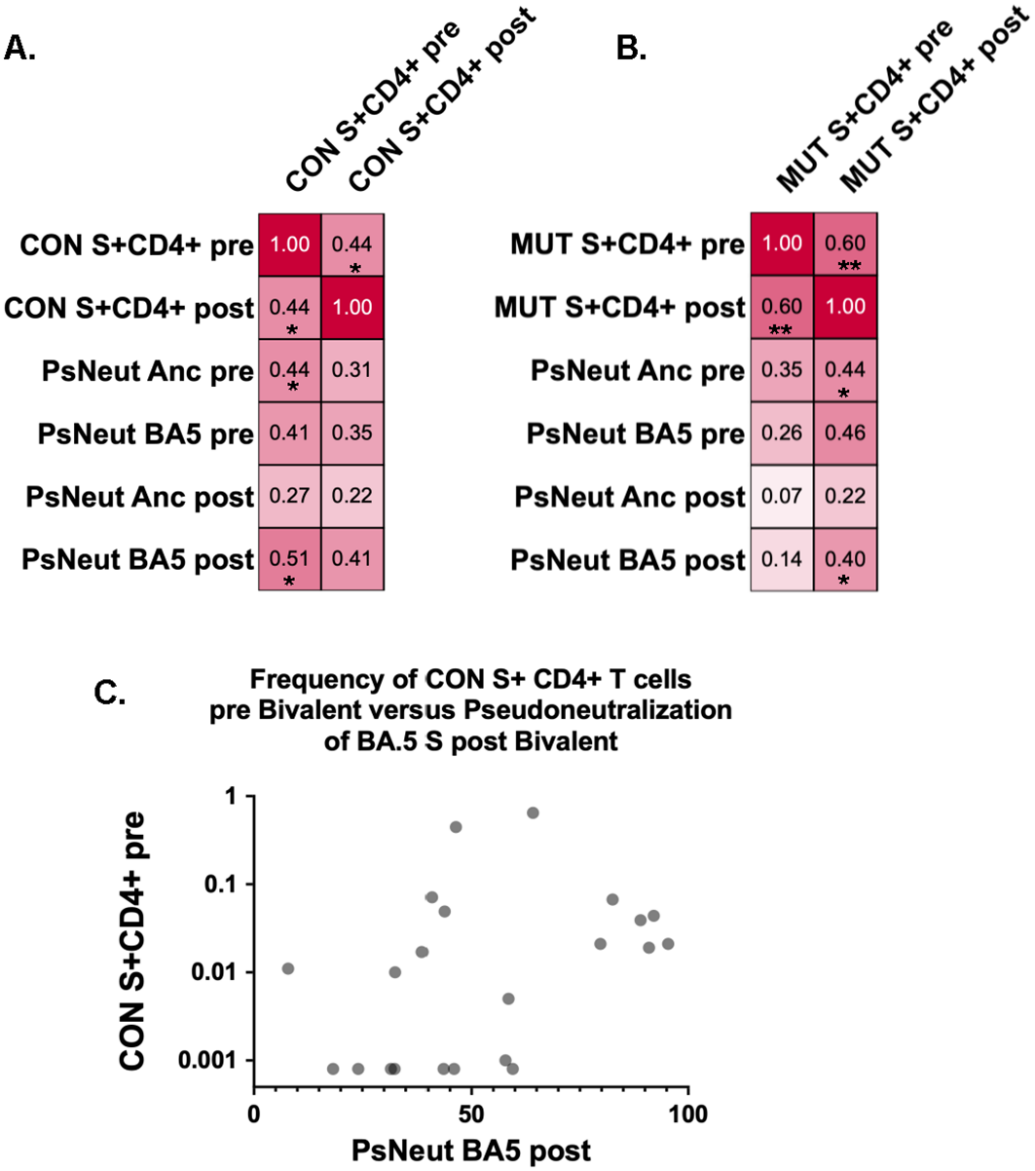
Polyfunctional Conserved and Omicron mutated S-specific CD4 T cells correlate with antibody neutralization of variant spike ACE2 binding. **(A)** Spearman correlation plot of conserved (CON) S-specific CD4^+^ T-cell frequencies and %ACE2 inhibition of ancestral spike (PsNeut Anc) and of BA.5 spike (PsNeutBA5) for pre- and post-bivalent time points. *p < 0.05, **p < 0.01. **(B)** Spearman correlation plot of Omicron mutated (MUT) S-specific CD4^+^ T-cell frequencies and %ACE2 inhibition of ancestral spike (PsNeut Anc) and of BA.5 spike (PsNeutBA5) for pre- and post-bivalent time points. **(C)** Multivariate plot for the frequency of cytokine-positive memory CD4 T cells in response to conserved spike peptides pre-bivalent and antibody pseudo neutralization of BA.5 S post-bivalent.

Repeated boosting with monovalent vaccines and bivalent vaccinations were recommended in the immunocompromised population due to lower rates of seroconversion (23–27, 40, 54). CD4 T cells play key roles supporting B cells and antibody responses (43, 62). Therefore, we sought to investigate how CON- and MUT-specific CD4 T-cell responses correlated to antibody neutralization of both ancestral and BA.4/5 S to understand how T-cell responses may contribute to effective vaccine strategies. Post-bivalent vaccination, there was a moderate correlation between CON S- and MUT S-specific CD4 T cells with BA.5 antibody pseudo-neutralization that was nearly identical (Spearman coefficient *r* = 0.41 and *r* = 0.40, respectively) (**Figures 4A, B**). Interestingly, higher frequencies of CON S- but not MUT S-specific CD4 T-cell responses pre-bivalent vaccination significantly positively correlated with increased BA.5 antibody pseudo-neutralization post-bivalent vaccination (**Figures 4A, C**). CON S-specific CD4 T-cell frequencies were not significantly correlated with Anc S antibody pseudo-neutralization, likely because most SOTRs in our cohort had high levels of S pseudo-neutralization post-bivalent vaccination (**Supplementary Figure 8B**). Significant Spearman correlations are summarized in **Table 2**.

**Table 2:** Summary of significant *p*-values for Spearman correlations.

Correlations with S-specific CD4 T cells			
Measurement	Compared to	Spearman *r*	*p*-values
Frequency CON S-specific CD4 T cells PRE	Frequency CON S-specific CD4 T cells POST	0.44	0.038
	Antibody PsNeut of Anc S PRE	0.44	0.041
	Antibody PsNeut of BA.4 S POST	0.51	0.015
Frequency MUT S-specific CD4 T cells POST	Frequency MUT S-specific CD4 T cells PRE	0.6	0.003
	Antibody PsNeut of Anc S PRE	0.44	0.038
	Antibody PsNeut of BA.4 S POST	0.40	0.033

Lastly, the frequencies of CON S- and MUT S-specific CD4 T-cell responses pre-bivalent significantly and positively correlated with the respective frequencies measured post-bivalent vaccination (**Figures 4A, B**). Therefore, we examined characteristics, including the organ transplanted, number of previous vaccines, age, and triple immunosuppression status of SOTRs, between those with (Responders) and those without (Non-responders) MUT S responses pre-bivalent vaccination to determine whether these MUT S “Non-responders” have any common characteristics (**Table 3**). No characteristics were significantly different between Responders and Non-responders except that kidney transplantation was more common and lung transplantation was less common in Responders (**Table 3**).

**Table 3:** Cytokine-producing CD4 T cells in response to Omicron-specific peptides PRE BIVALENT.

# Non-responders (%)	8 (36.4%)	# Responders (%)	14 (63.6%)
Organ (%)		Organ (%)	
Lung	2/8 (25%)	Lung	1/14 (7.1%)
Kidney	3/8 (37.5%)	Kidney	9/14 (64.3%)
Liver	2/8 (25%)	Liver	3/14 (21.4%)
Two organs	1/8 (12.5%)	Two organs	1/14 (7.1%)
Triple immunosuppressives (%)	4/8 (50%)	Triple immunosuppressives (%)	6/14 (42.8%)
Median age (range)	67.5 (35, 80)	Median age (range)	57.5 (35, 80)
Median # of vaccines (range)	5.5 (4, 6)	Median # of vaccines (range)	5 (4, 8)

Thus, CD4 T-cell responses to both conserved and Omicron mutated sequences correlated with increased pseudo-neutralization of BA.5 S. Furthermore, CON-specific CD4 T-cell responses correlated with BA.5 pseudo-neutralization even prior to the bivalent vaccination, and higher frequencies of CON S-specific T cells prior to bivalent vaccination significantly correlated with increased BA.5 S pseudo-neutralization post-bivalent. These data, in addition to the significant and positive correlation between pre- and post-bivalent S-specific CD4 T-cell frequencies, indicate that pre-vaccine immune status is important. CD4 T-cell responses generated pre-bivalent vaccination in response to repeated monovalent boosting play an important role in subsequent antibody development.

### 2.6 S-specific CD4 T-cell responses to conserved and Omicron mutated S sequences do not differ between SOTRs and healthy controls

**Figure 5:**
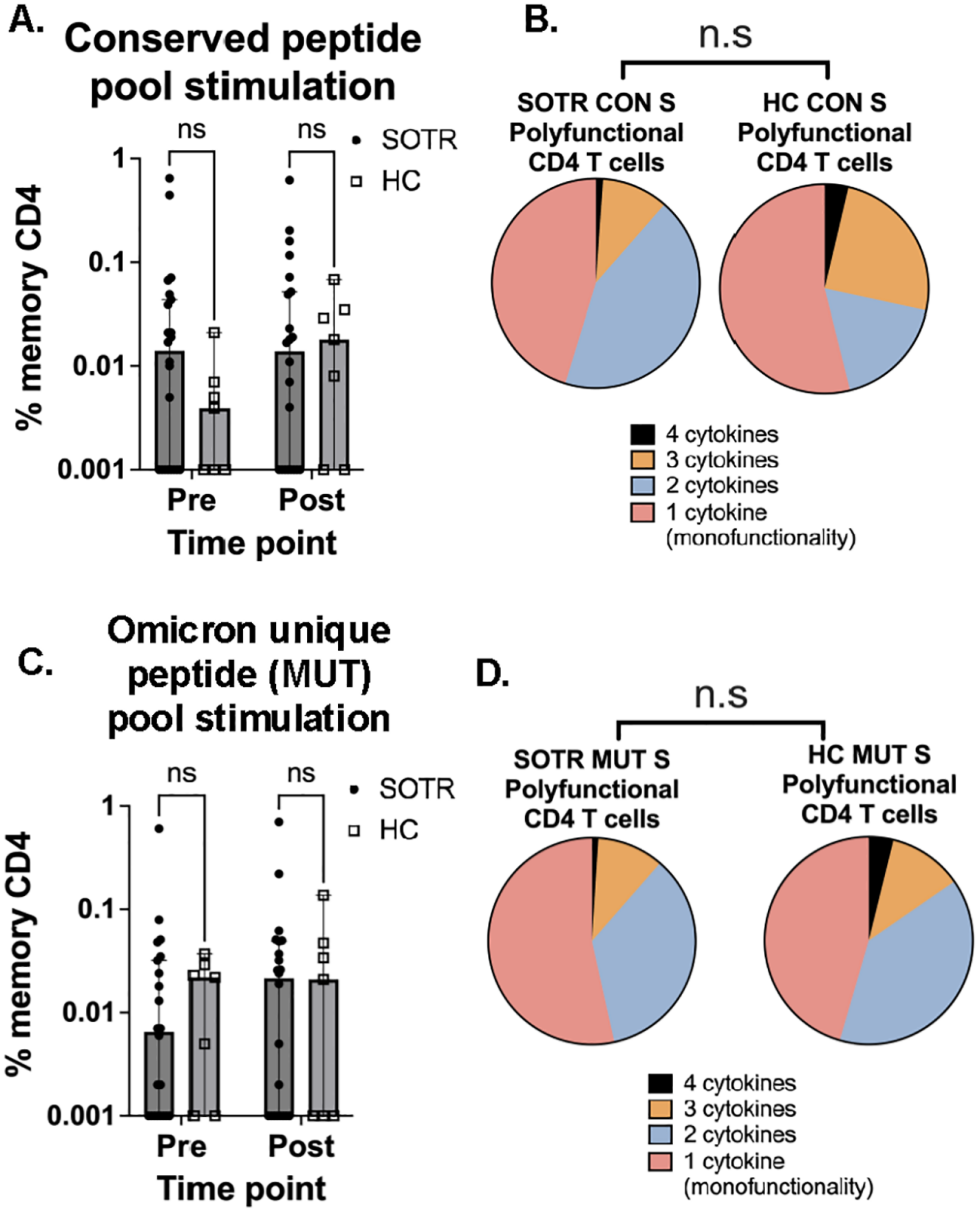
Repeated vaccination in SOTRs results in similar polyfunctionality to healthy individuals post-bivalent vaccination. **(A)** Frequency of bulk cytokine-producing memory CD4 T cells in response to CON peptide stimulation pre- and post-bivalent shown with median and 95% confidence intervals. Statistics tested with Mann–Whitney test with Holm–Sidak’s multiple comparisons. **(B)** Pie charts showing the summarized fraction of polyfunctional cytokine response comprising any four, three, two, or one cytokine from CD4 T cells stimulated with conserved S (CON) peptides. Significance tested with multiple Mann–Whitney tests with Holm–Sidak’s multiple comparisons. **(C)** Pie charts showing the summarized fraction of polyfunctional cytokine response comprising any four, three, two, or one cytokine from CD4 T cells stimulated with Omicron mutated S (MUT) peptides. Significance tested with multiple Mann–Whitney tests with Holm–Sidak’s multiple comparisons. **(D)** Frequency of bulk cytokine-producing memory CD4 T cells in response to MUT peptide stimulation pre- and post-bivalent shown with median and 95% confidence intervals. Statistics tested with Mann–Whitney test with Holm–Sidak’s multiple comparisons.

To determine how pharmacologic immunosuppression may be impacting the S-specific CD4 T-cell responses in SOTRs, S-specific CD4 T-cell responses from SOTRs post-bivalent immunization were compared to responses in non-SOTR healthy controls (HCs). Frequencies of cytokine-producing CD4 T cells (**Figure 5A**) and polyfunctionality for any four, three, two, or one cytokine (**Figure 5B**; **Supplementary Figure 9A**) in response to CON S were not significantly different between SOTRs and HCs pre- and post-bivalent vaccination.

Cytokine frequencies within MUT S-specific responses in HCs were also compared to the frequencies in SOTRs post-bivalent vaccination, with no significant difference in the frequencies of cytokine-producing CD4 T cells demonstrated (**Figure 5C**). Similarly to CON S responses, there are no significant differences between frequencies of MUT S-specific T cells polyfunctional for any four, three, two, or one cytokine between HCs and SOTRs post-bivalent vaccination (**Figure 5D**; **Supplementary Figure 9B**). These data suggest that repeated boosting in SOTRs increased their CD4 response to SARS-CoV-2 to levels similar to those of healthy individuals. However, the number of SOTRs in our study with low T-cell responses after boosting was small and larger cohorts comparing SOTRs to HC may reveal important differences in post-bivalent responses.

## 3 Discussion

Bivalent mRNA vaccines have provided the opportunity to investigate memory responses to evolving antigens, particularly in populations at high risk of infectious diseases, such as SOTRs. Post-bivalent immunization, BA.4/5-stimulated CD4 T-cell responses were characterized by IFNγ and TNF mono- and polyfunctionality with an activated effector phenotype compared to Anc stimulation. We further investigated the role of sequence differences between Anc and BA.4/5 S on this polarity. Collectively, we demonstrated that T cells cross-reactive to sequences containing Omicron mutations existed prior to bivalent vaccination, bivalent boosting elicited similar responses regardless of whether the T cell recognized conserved or Omicron mutated sequences, and T cells specific for conserved and Omicron mutated S post-bivalent vaccination correlated with pseudo-neutralization of Omicron-specific S proteins.

The detection of Omicron mutated S-specific CD4 T cells prior to the bivalent vaccine and without evidence of infection suggests that ancestral mRNA vaccines generated cross-reactive responses. Previous work has shown that T-cell responses against SARS-CoV-2 were specific for conserved regions between SARS-CoV-2 variants (56–60). Tarke et al. (2022) showed that the majority of S-specific CD4 responses generated post monovalent vaccination could respond to Omicron S peptide stimulation using peptide pools generated in the same fashion as those used in this study (56). Moreover, T-cell responses induced by monovalent mRNA vaccines were reported to respond similarly to ancestral and Omicron spike stimulation, even in SOTR populations (27, 54, 57, 58). That hospitalizations were lower despite drastic evolution in the Omicron variant spike RBD mediating neutralizing antibody evasion supports that T cells may provide some protection from severe disease (61). Importantly, Tarke et al. (2022) also demonstrated that most known T-cell epitopes within S do not contain Omicron-specific a.a. mutations. Therefore, even when studying T-cell responses to overlapping peptide pools from Omicron S sequences, the responses are likely to conserved sequences. Our data corroborate these findings in SOTRs by showing that the quantitative differences in polyfunctionality observed in response to the full-length BA.4/5 S peptide pool used for **Figures 1**, **2** may reflect the recognition of cross-reactive and conserved responses rather than *de novo* Omicron-specific responses.

The positive correlation between S-specific CD4 T-cell magnitude and anti-S pseudo-neutralization is further evidence that SOTRs generate CD4 T-cell help to respond to novel antigens, as previously reported in non-SOTRs in SARS-CoV-2 infection (56–58). The relationship between CD4 T cells and productive antibody responses has been characterized in infections with other highly diverse viruses, such as influenza and HIV (62–64). Importantly, our data suggest that pre-existing CD4 T-cell responses to conserved S sequences correlated with neutralization of highly mutated variant S in SOTRs, consistent with data in other infections. In the context of influenza, CD4 T cells in immunocompetent individuals exposed to circulating H1N1 produced cytokines in response to pandemic H1N1 hemagglutinin (65). Our data support that SOTRs also respond to variant antigens by mobilizing previously generated cross-reactive and conserved CD4 T-cell responses without the need for variant-specific immunization, as has been previously reported in healthy individuals (28). Interestingly, COVID-19 vaccine data from other immunocompromised populations, such as in IBD, have shown that T-cell responses are of a similar magnitude to HCs even when antibody responses are still relatively decreased (66). Therefore, these data suggest SOTRs and other immunocompromised populations need even more repeated antigen exposure to engage these mechanisms, both at the CD4 T cell and antibody levels.

Lastly, we did not observe differences in the magnitude of S-specific CD4 T-cell responses in SOTRs versus HCs post-bivalent vaccination. We hypothesize that this is due to SOTRs in our cohort having received four or five doses of ancestral COVID-19 vaccines prior to receipt of the bivalent booster vaccine. The response after repeated, increased boosting in SOTRs is an important observation considering Boyarsky et al. reported that 46% of their SOTR cohort did not generate an antibody response after dose 1 or 2 of the original monovalent vaccines (24). Additional data from our SOTR cohort further implicates the necessity of boosting for the durability of these responses, even after a bivalent boost (67). These data demonstrate the importance of continuing to study vaccine responses in SOTRs longitudinally to understand how to best time and dose boosting.

Limitations of this study include that CD8 T cells were not detected by cytokine expression and thus not assessed. We did not test T-cell responses against minimally recognized epitopes from the Omicron S sequence. In addition, the numbers of SOTRs and HCs were low, negating the ability to completely control for sex, age, previous reported infections, or transplanted organ. Studies with larger cohorts are needed to confirm whether the SOTR T-cell responses are similar to HCs after multiple vaccine doses. However, the small numbers were sufficient to find significant associations between CD4 T cells and antibody responses in SOTRs. Furthermore, the number of previous vaccines, age, and triple immunosuppression status of SOTRs did not differ between those with and without Omicron mutated S CD4 T-cell responses pre-bivalent vaccination.

In conclusion, additional doses of vaccine for SOTRs not only improved antibody response but also enhanced the magnitude of the CD4 T-cell response. While reformulation of vaccines to match circulating variants may not be as critical for the T-cell response, additional doses of vaccines with conserved or cross-reactive T-cell epitopes are likely important for generating effective T-cell responses to novel pathogens in SOTRs.

## 4 Methods

### 4.1 Sex as a biological variable

Male and female SOTRs were eligible for recruitment. Samples from both sexes were used in this study.

### 4.2 Statistical analysis

Statistical analyses were conducted using GraphPad Prism v9 and v10 and SPICE 6 v6.1. Statistical tests used in individual figures are noted in figure legends. Significance was determined for two-sided *p*-values less than 0.05.

### 4.3 Study approval

SOTR participants were enrolled in a national prospective, observational cohort: “COVID-19 antibody testing of recipients of solid organ transplants and patients with chronic diseases”, which was approved by the Johns Hopkins IRB00248540, as previously described (40). All SOTR participants were recruited virtually and provided detailed transplant history as well as oral informed consent (waiver of written consent granted). All vaccines were also administered independently in the community. The cohort consisted of participants who did not have a substantial antibody response after the two-dose series. For this cohort, blood samples were obtained before 2 weeks after bivalent vaccine. HC participants were enrolled under Johns Hopkins IRB 00249350, and all received bivalent SARS-CoV-2 mRNA vaccine. Available PBMCs from 2 to 4 weeks post-bivalent vaccination were used in this study. All HCs provided written informed consent and contributed limited demographic data (e.g., sex, race, and decade of age).

### 4.4 Peripheral blood mononuclear cell preparation

Blood was collected in acid citrate dextrose tubes, and plasma was isolated by centrifugation and stored at –80°C until further analysis. PBMCs were isolated within 24 h of blood collection, as previously described (68). Aliquoted PBMCs were stored in liquid nitrogen freezers until thawing for further studies.

### 4.5 ACE2 inhibition surrogate neutralization assay

The Meso Scale Discovery (MSD) Angiotensin Converting Enzyme-2 (ACE2) inhibition assay was used to measure the inhibition of ACE2 receptor binding to the S protein (% ACE2 inhibition) as previously described (25). All samples were assayed on MSD V-PLEX SARS-CoV-2 panels 27 and 34 at a dilution of 1:100. Plates were read on MSD QuickPlex SQ 120, and percentage inhibition was calculated using the manufacturer’s protocol. Statistical analyses were conducted using GraphPad Prism v9 and v10.

### 4.6 Antigen recall assay

An antigen recall assay was utilized to assess S antigen-specific T-cell cytokine production following *in vitro* restimulation as previously described (54). PBMCs were thawed using a CryoThaw adaptor (69) (Medax) into 10 mL of RPMI (Gibco) with 10% fetal bovine serum (FBS) (Atlanta Biologicals). PBMCs rested for approximately 6 h following thaw. After the rest, 1e6 cells were cultured in 200 mL of RPMI supplemented with 10% FBS in a 96-well plate. Cells were stimulated with 1 μg/mL ancestral (PM-WCPV-S-1), Omicron BA.4/5 (PM-SARS2-SMUT10-1) (JPT Peptide Technologies), conserved or Omicron mutated (La Jolla Institute for Immunology) SARS-CoV-2 S peptide pools that were suspended in dimethyl sulfoxide (DMSO) in the presence of 10 μg/mL of brefeldin A (Millipore Sigma) overnight (approximately 14 h). The JPT Peptide Technologies peptide pools contain 315 15mers with an 11-a.a. overlap, that span the entire SARS-CoV-2 spike protein. The peptide pools from La Jolla Institute for Immunology contain 64 peptides with an 11-a.a. overlap, which span the regions of spike protein that are mutated for BA.4/5 SARS-CoV-2 and were synthesized as previously described (56). The BA.4/5 peptides encompass specific mutations of SARS-CoV-2, including T19I, L24del, P25del, P26del, A27S, H69del, V70del, G142D, V213G, G339D, S371F, S373P, S375F, T376A, D405N, R408S, K417N, N440K, L452R, S477N, T478K, E484A, F486V, Q498R, N501Y, Y505H, D614G, H655Y, N679K, P681H, N764K, D796Y, Q954H, and N969K. Unstimulated wells were supplemented with equivalent volume DMSO and brefeldin A for all samples. The following day, surface and intracellular staining was performed for flow cytometry. All samples had individual unstimulated conditions (DMSO only) and stimulated conditions (ancestral or BA.5) and were all background subtracted. If a sample was negative or 0, it was changed to the lowest detectable value on the day the samples were run. These were considered a nonresponse. Peptides were prescreened for background activity in pre-pandemic samples and determined to have background activity comparable to DMSO-only controls.

### 4.7 Flow cytometry staining

Antibodies used for phenotypic and metabolic analyses by flow cytometry are listed in **Supplementary Table 1** and as previously reported (54, 68). Cells were washed once in PBS and immediately stained for viability with Invitrogen Live/Dead Fixable Blue Dead Cell Stain for 10 min at room temperature. Cell surface staining was performed in 50 μL of 20% BD Horizon Brilliant Stain Buffer + PBS with surface stain Ab cocktail for 20 min at room temperature. Cells were fixed and permeabilized with eBioscience FoxP3/Transcription Factor Staining Buffer Set with 1× Fixation/Permeabilization reagent for 20 min at room temperature. Cells were washed with 1× Permeabilization/Wash buffer. Intracellular staining (ICS) was performed in 50 μL of 1× Permeabilization/Wash buffer with ICS Ab cocktail for 20 min at room temperature. Cells were washed once with Permeabilization/Wash buffer, then resuspended in 1% paraformaldehyde. Samples were run on a 4-laser Cytek Aurora spectral flow cytometer.

### 4.8 Flow cytometry analysis

FCS files were analyzed using FlowJo v10.9.0 software using manual gating and plugins for UMAP (v3.3.3), Xshift (v1.4.1), AutoGateCategorical (v2.6.1), and ClusterExplorer (v1.7.6). Cytokine and polyfunctional category frequencies were background subtracted using frequencies measured in unstimulated conditions. Frequencies of clusters identified by Xshift were applied to individual samples, and frequency of total population was determined. These frequencies were then stratified according to the indicated parameters and analyzed in GraphPad Prism v9 and v10. Polyfunctional T-cell responses were analyzed using Pestle v2.0 and SPICE 6 v6.1 (70). Polyfunctional cytokine frequencies are shown on the log-transformed scale. Zero values were set to the lowest detected value 0.0001 for the log-transformed axis.

## Data Availability

The raw data supporting the conclusions of this article will be made available by the authors, without undue reservation.
