# Supplementary material for: Conserved CD4 T-cell responses correlate with antibody neutralization in solid organ transplant recipients after bivalent SARS-CoV-2 vaccination
Source: Front Immunol. 2026 Jul 24;17:1856861. doi: 10.3389/fimmu.2026.1856861 (PMC13447162; doi:10.3389/fimmu.2026.1856861)
Supplement: Supplementary file 1 [file DataSheet1.pdf]

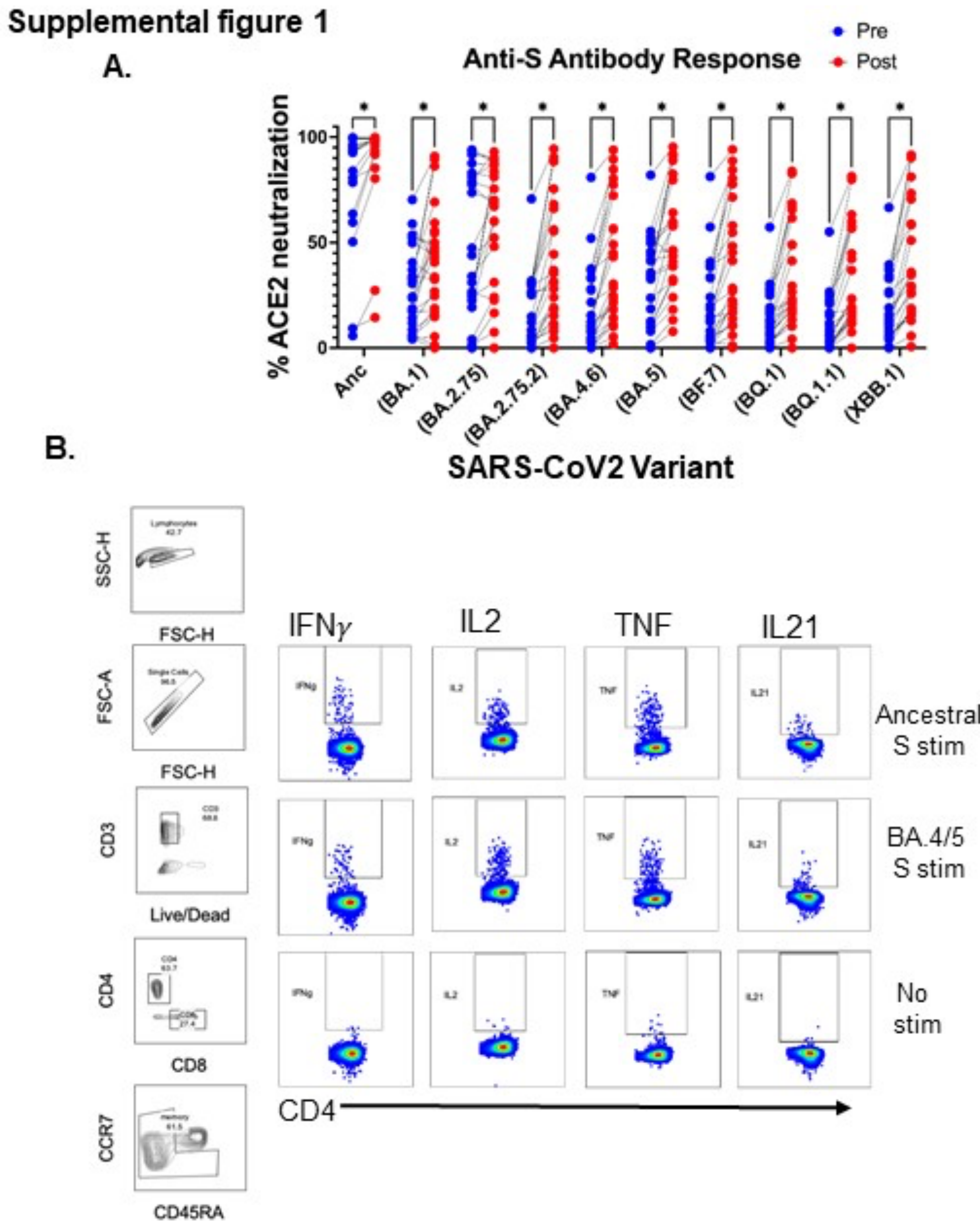

Supplemental Figure 1 A) Percent inhibition of Spike binding ACE2 (% ACE2 inhibition) by SOTR plasma pre and post bivalent vaccination. Percent ACE2 inhibition was measured for multiple SARS-CoV-2 variants. Anc = Ancestral Significance tested using Multiple Wilcoxon tests. *$p < 0.05$ B) Representative gating for cytokine producing (positive) S-specific CD4+ T cells.

Supplemental figure 2

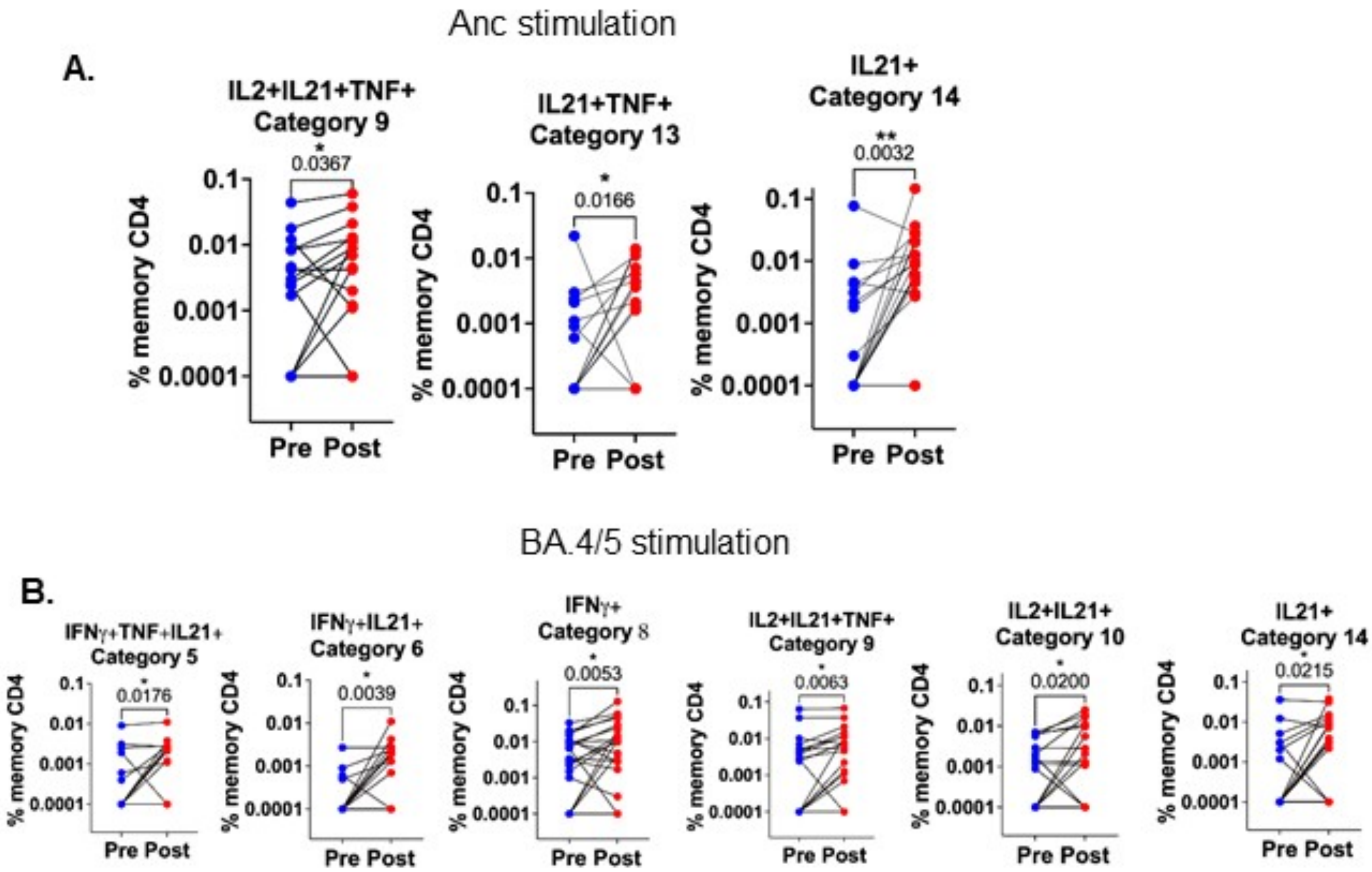

Supplemental Figure 2 A) Polyfunctional cytokine categories that are statistically significantly increased post bivalent vaccine in response to overlapping Ancestral spike (Anc) peptides. Significance tested with Wilcoxon matched pairs signed rank test. B) Polyfunctional cytokine categories that are statistically significantly increased post bivalent vaccine in response to overlapping BA.4/5 spike peptides. Significance tested with Wilcoxon matched pairs signed rank test.

Supplemental figure 3

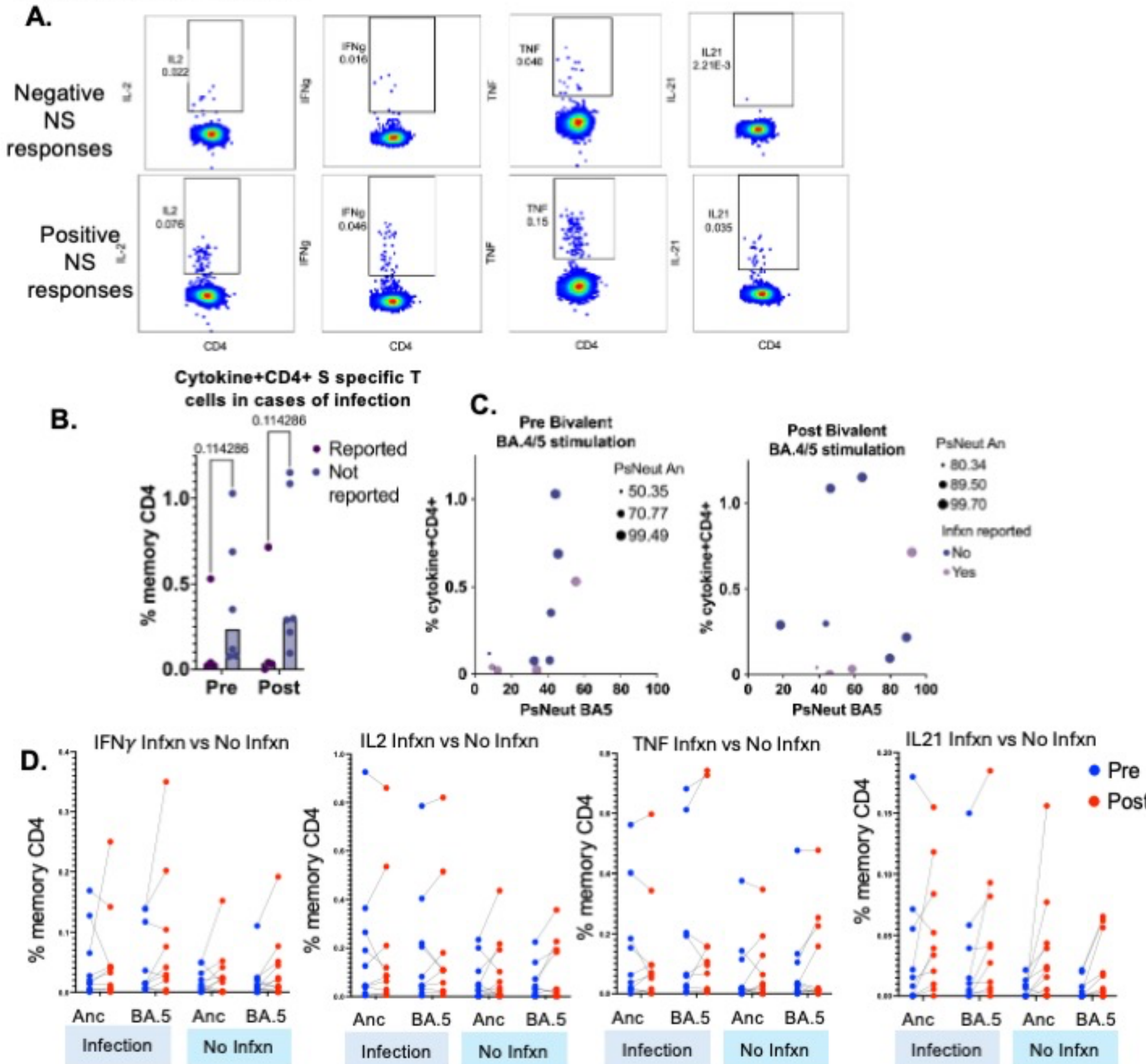

Supplemental Figure 3 A) Representative flow cytometry plots for NS positive responses by CD4 T cells. B) Frequency of BA.4/5 S-specific CD4+ T cell stratified by reported breakthrough infection. Significance tested with multiple Mann-Whitney tests. C) Multivariate analysis of frequency of BA.4/5 S-specific CD4+ T cells correlated with % ACE2 neutralization (PsNeut) of ancestral and BA.5 lineage Spike by matched plasma. D) Frequency of memory CD4+ T cells producing cytokines (TNF, IFN-γ, IL-2, or IL-21) stratified by T cell assay evidence of previous infection. Sample paired comparison of CD4+ T cell responses recalled by overlapping Ancestral (Anc) or BA4.5 spike peptides.

Supplemental Figure 4

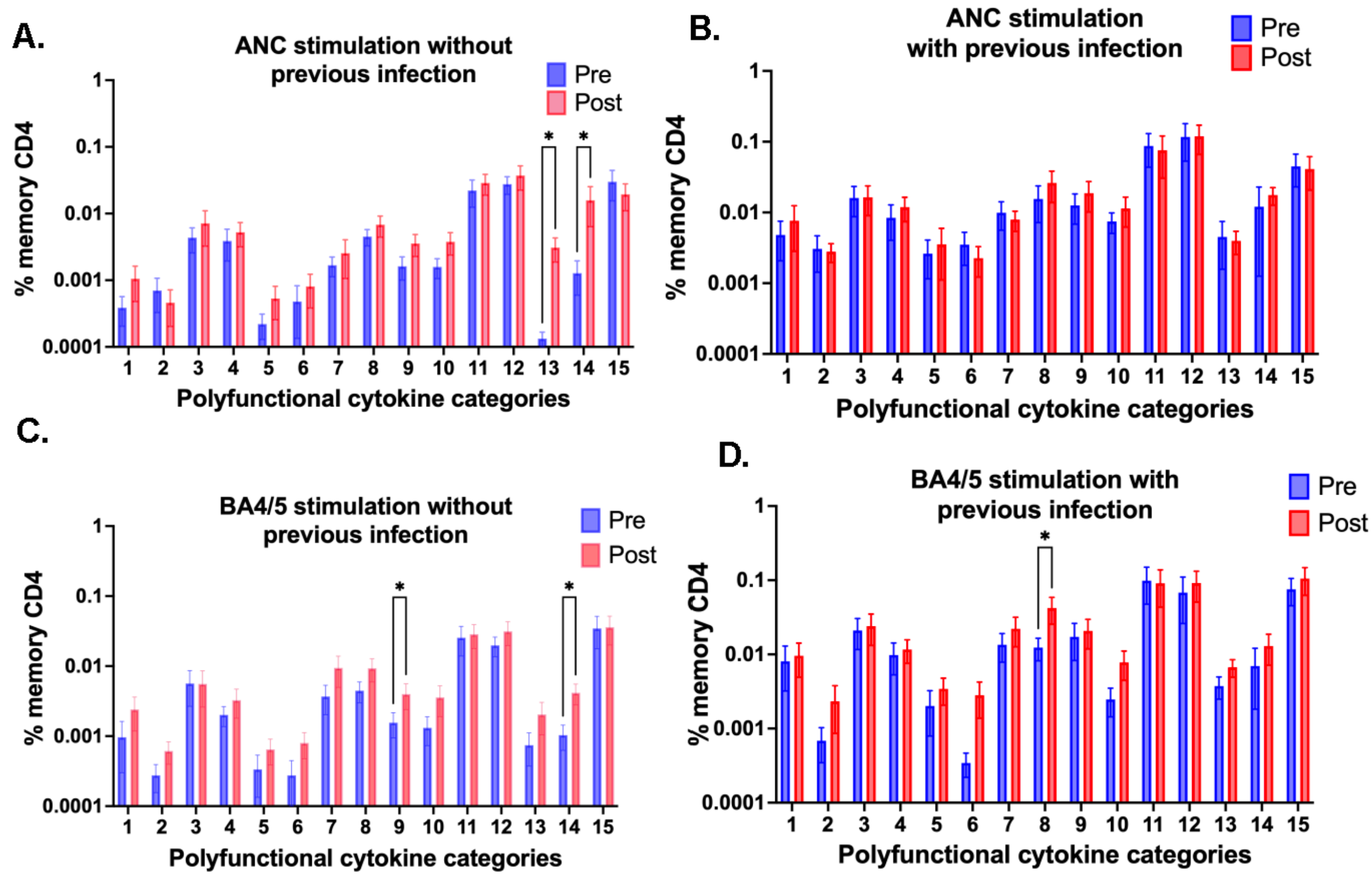

Supplemental Figure 4 A) Frequency of Polyfunctional cytokine categories in memory CD4+ T cells in response to Ancestral spike overlapping peptide pools without previous infection. B) Frequency of Polyfunctional cytokine categories in memory CD4+ T cells in response to overlapping Ancestral spike peptide pools (Anc) with evidence previous infection. C) Frequency of Polyfunctional cytokine categories in memory CD4+ T cells in response to overlapping BA.4/5 spike peptide pools (BA.4/5) without previous infection. D) Frequency of Polyfunctional cytokine categories in memory CD4+ T cells in response to overlapping BA.4/5 spike peptide pools with evidence of previous infection. Significance tested with Wilcoxon matched pairs signed rank test. *$p < 0.05$.

Supplemental figure 5

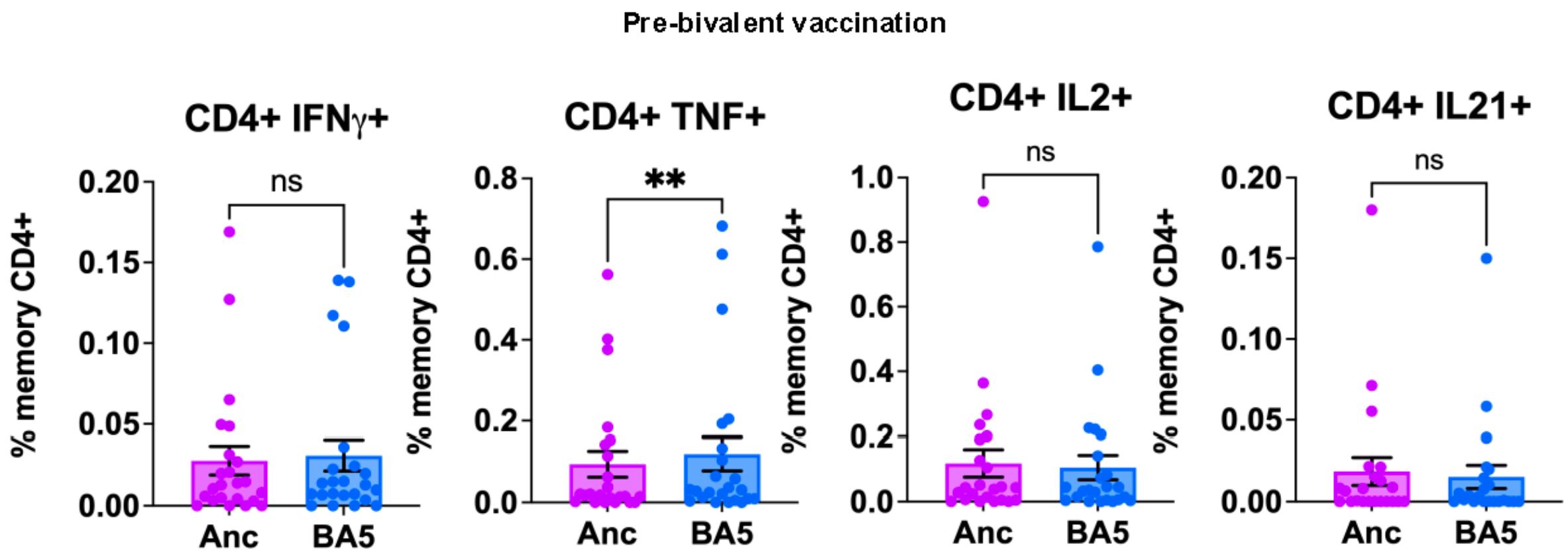

Supplemental Figure 5 A) Frequency of memory CD4+ T cells producing cytokines (TNF, IFN-γ, IL-2, or IL-21) in response to ancestral (Anc) or BA.4/5 peptides pre bivalent vaccination. Sample paired comparison of CD4 responses recalled by Anc or BA.4/5 S peptides. All data shown as Mean and SEM.

Supplemental Figure 6

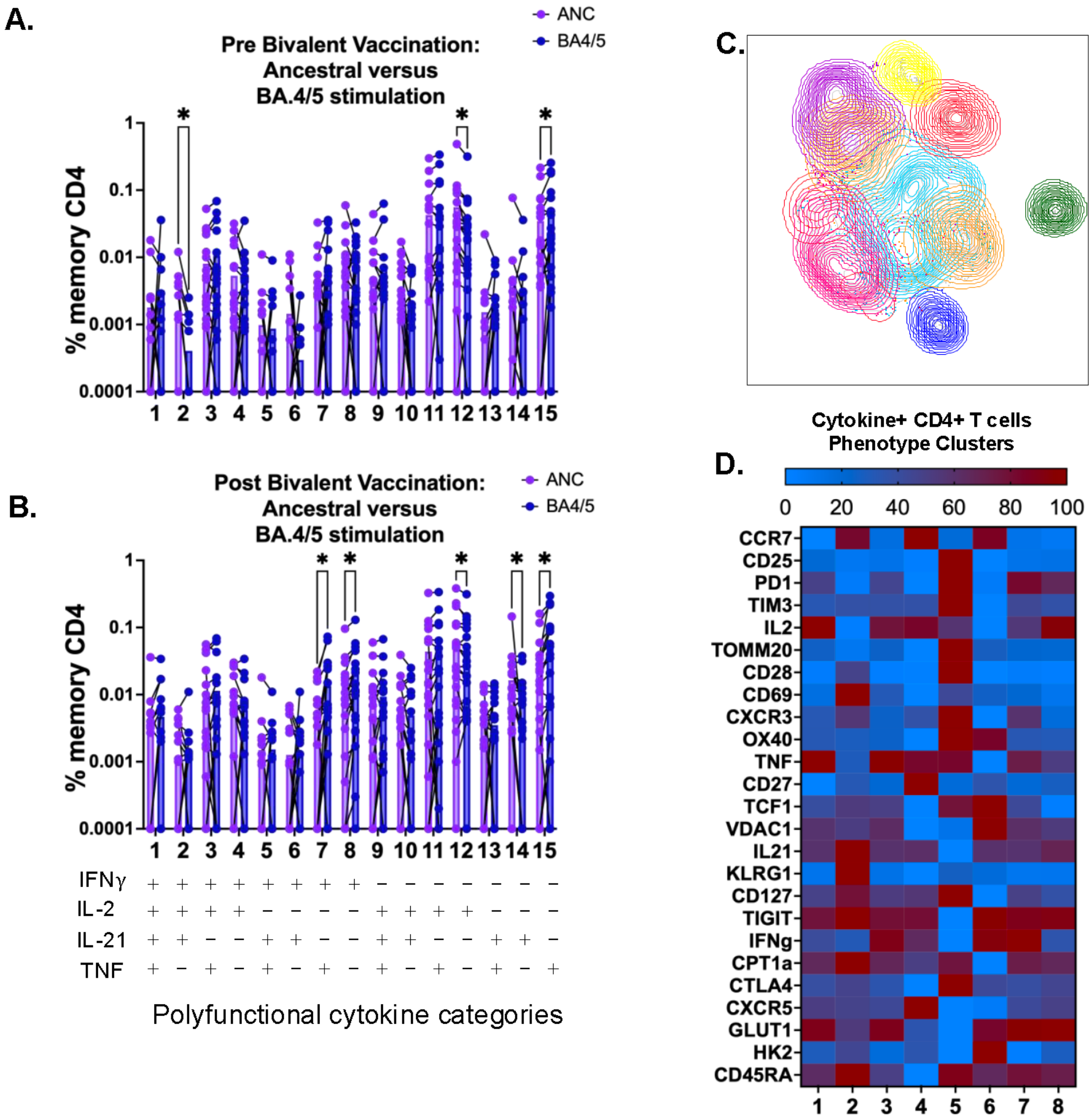

Supplemental Figure 6 A) Frequency of Polyfunctional cytokine categories in memory CD4+ T cells pre bivalent vaccination. B) Frequency of Polyfunctional cytokine categories in memory CD4+ T cells post bivalent vaccination. C) UMAP projection of S specific CD4 T cells with overlayed phenotype clusters. D) Heatmap of normalized MFIs for markers expressed in defined clusters. Significance tested with Wilcoxon matched pairs signed rank test.

Supplemental Figure 7

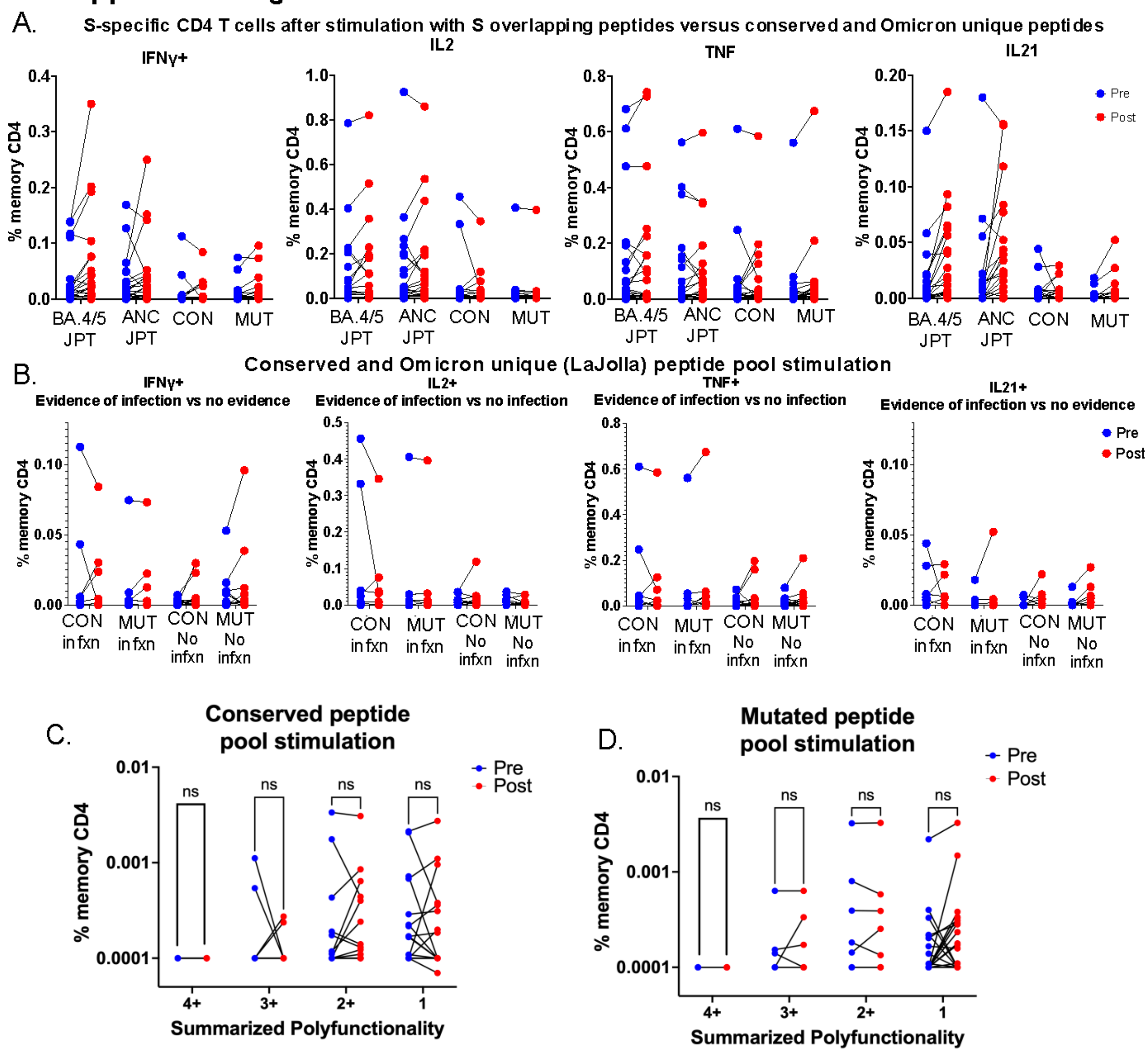

Supplemental Figure 7 A) Frequency of memory CD4+ T cells producing cytokines (TNF, IFN-γ, IL-2, or IL-21) stratified by peptide pool. Sample paired comparison of CD4+ T cell responses recalled by Ancestral, Omicron, conserved (CON), Omicron mutated sequences (MUT) spike peptides pre and post bivalent. B) Frequency of memory CD4+ T cells producing cytokines (TNF, IFN-γ, IL-2, or IL-21) stratified by evidence of previous infection. Sample paired comparison of CD4+ T cell responses recalled by conserved (CON) and Omicron mutated (MUT) spike peptides. C) Frequencies of polyfunctional cytokine categories in memory CON S-specific CD4+ T cells pre and post bivalent vaccination. D) Polyfunctional cytokine categories that are statistically significantly different post bivalent vaccine in response to MUT spike peptides. Significance tested with Wilcoxon matched pairs signed rank test.

Supplemental Figure 8

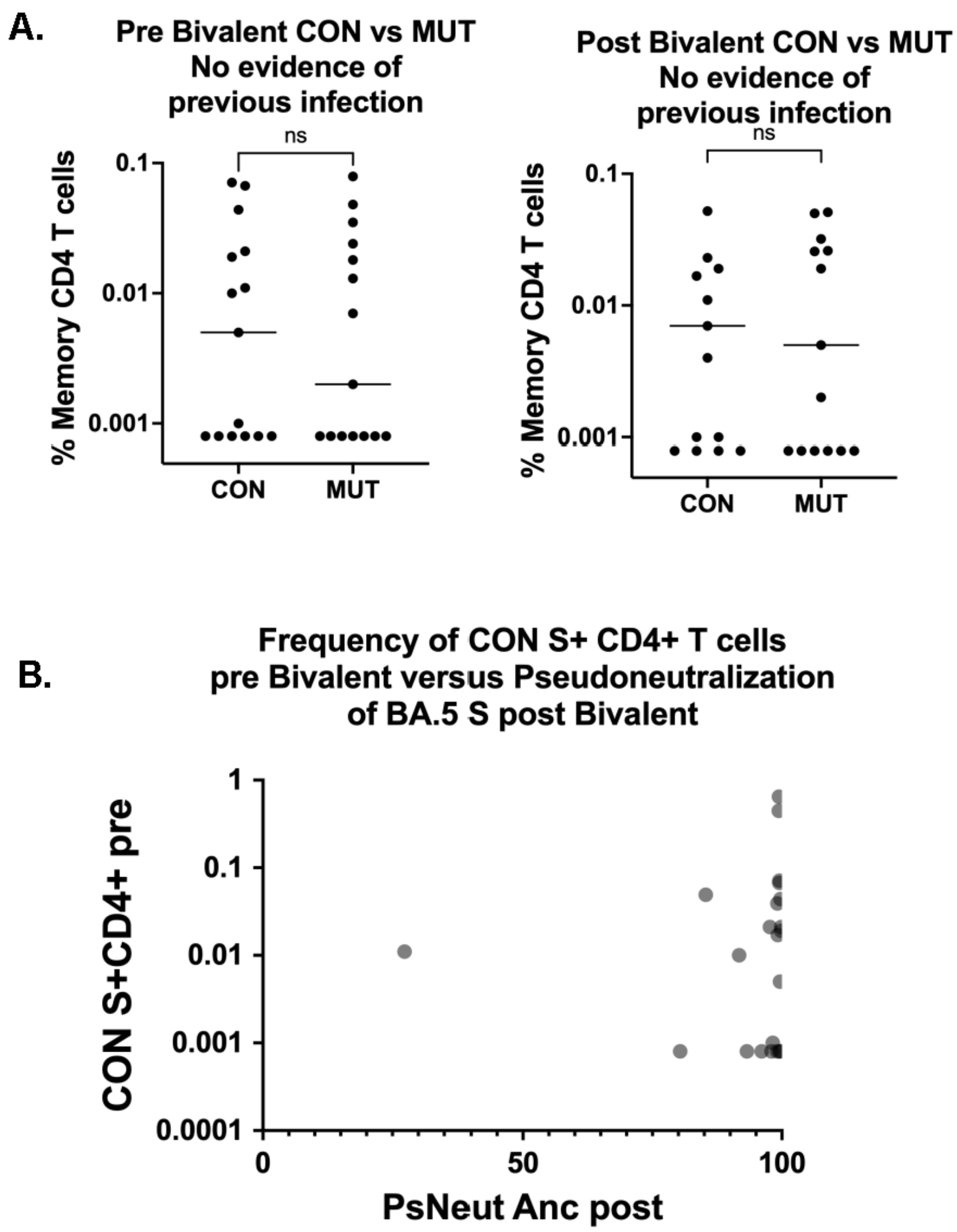

Supplemental Figure 8 A) Frequency of CON versus MUT specific CD4 T cells at the pre and post bivalent time points. Significance tested with Wilcoxon matched pairs signed rank test. B) Multivariate plot for the frequency of cytokine positive memory CD4 T cells in response to conserved spike peptides pre bivalent and antibody pseudo neutralization of Anc S post bivalent.

Supplemental Figure 9

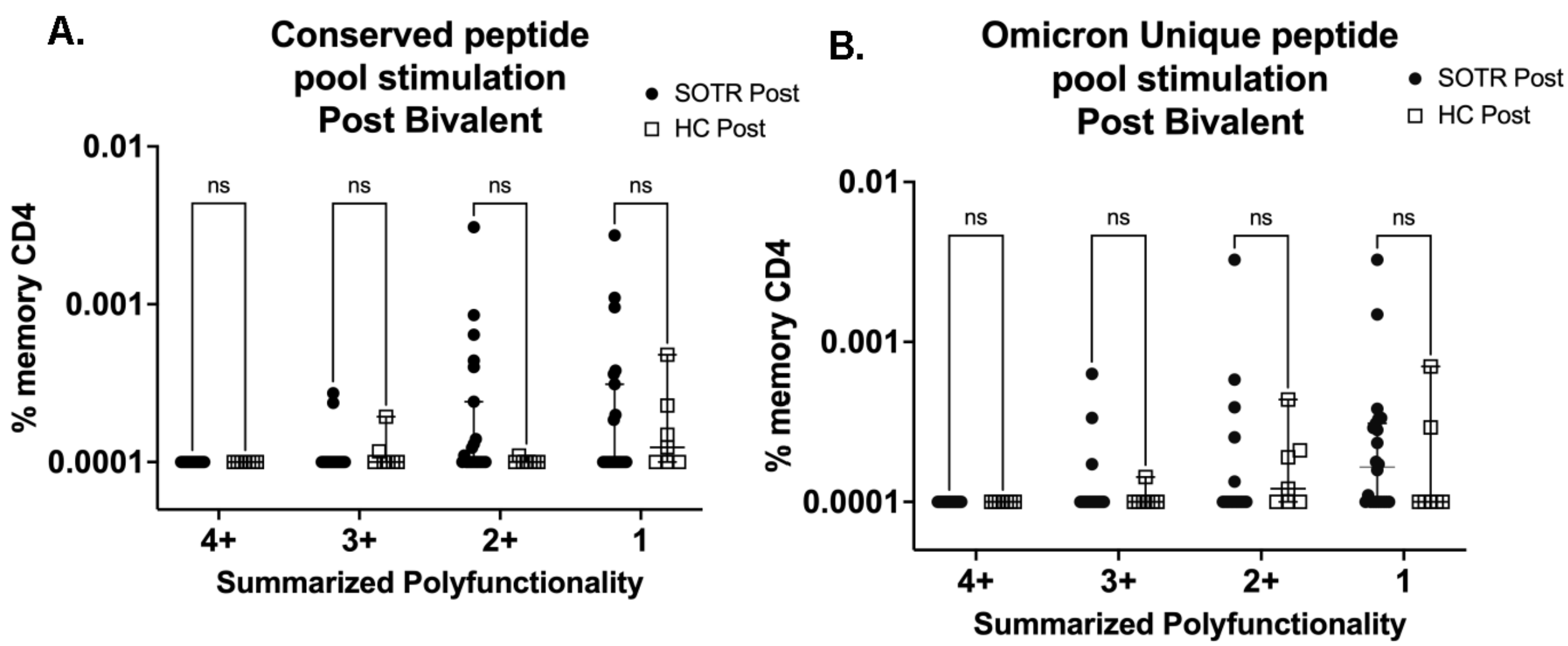

Supplemental Figure 9 A) Frequency of conserved S (CON) stimulated CD4 T cells expression 4, 3, 2 or 1 cytokines in any combination post bivalent. Statistics tested with Mann-Whitney test with Holm-Sidak's multiple comparisons. B) Frequency of Omicron mutated (MUT) S stimulated CD4 T cells expression 4, 3, 2 or 1 cytokines in any combination post bivalent. Statistics tested with Mann-Whitney test with Holm-Sidak's multiple comparisons.
